# Effect of Instrumented Spine Surgery on Length of Stay

**DOI:** 10.5435/JAAOSGlobal-D-22-00231

**Published:** 2023-05-15

**Authors:** Mary E. Lundgren, Alex N. Detwiler, Jeffery W. Lamping, Sarah L. Gael, Nai-Wei Chen, Rafid Kasir, James D. Whaley, Daniel K. Park

**Affiliations:** From the Department of Orthopedic Surgery, William Beaumont Hospital, Royal Oak, MI (Dr. Lundgren, Dr. Detwiler, Dr. Lamping, Dr. Gael, Dr. Kasir, Dr. Whaley, and Dr. Park), and the Beaumont Research Institute, Royal Oak, MI (Dr. Chen).

## Abstract

Total joint arthroplasty studies have identified that surgeries that take place later in the week have a longer length of stay compared with those earlier in the week. This has not been demonstrated in studies focused on anterior cervical diskectomy and fusions or minimally invasive lumbar laminectomies. All-inclusive instrumented spine surgeries, however, have not been analyzed. The purpose of this study was to determine whether day of surgery affects length of stay and whether there are predictive patient characteristics that affect length of stay in instrumented spine surgery.

**Methods:**

All instrumented spine surgeries in 2019 at a single academic tertiary center were retrospectively reviewed. Patients were categorized for surgical day and discharge disposition to home or a rehabilitation facility. Differences by patient characteristics in length of stay and discharge disposition were compared using Kruskal-Wallis and chi square tests along with multiple comparisons.

**Results:**

Seven hundred six patients were included in the analysis. Excluding Saturday, there were no differences in length of stay based on the day of surgery. Age older than 75 years, female, American Society of Anesthesiology (ASA) classification of 3 or 4, and an increased Charlson Comorbidity Index were all associated with a notable increase in length of stay. While most of the patients were discharged home, discharge to a rehabilitation facility stayed, on average, 4.7 days longer (6.8 days compared with 2.1 days, on average) and were associated with an age older than 66 years old, an ASA classification of 3 or 4, and a Charlson Comorbidity Index of 1 to 3.

**Conclusions:**

Day of surgery does not affect length of stay in instrumented spine surgeries. Discharge to a rehabilitation facility, however, did increase the length of stay as did age older than 75 years, higher ASA classification, and increased Charlson Comorbidity Index classification.

Length of hospital stay is one of the major drivers of total cost, in addition to the number of admission diagnoses and procedures, hospital size and region, patient income, fusion surgery, acute renal failure, sex, and coagulopathy.^1,2^ According to one study, length of stay increased the total cost after a spine procedure by 0.47% and a fusion increased the total cost by 69.89%.^1^ Efforts to identify modifiable factors that may decrease the length of stay and, therefore, decrease the total cost will be of benefit to an efficient and cost-effective healthcare system.

Several publications have indicated a difference in the length of stay based on the day the surgery took place for total joint arthroplasty procedures. There was only one study that indicated that there was no difference in the average length of stay for each day of the week for total joint arthroplasty,^3^ although most publications have contradicted this study. If the surgery took place earlier in the week (Monday, Tuesday, or Wednesday), there was a notable decrease in the length of stay compared with cases that took place later in the week (Thursday or Friday).^4,5,6,7,8,9,10^ This trend demonstrated in total joint arthroplasty has not been replicated in the all-spine literature. An anterior cervical diskectomy and fusion (ACDF) length of stay was not affected by the day of the week the surgery took place,^11^ and similarly, for a minimally invasive lumbar decompression, there was no difference in length of stay when comparing a surgery that took place early in the week and later in the week.^12^ A focused study on lumbar laminectomies for lumbar spinal stenosis highlighted that the location of discharge to a rehabilitation center not only was associated with an increased length of stay and higher healthcare cost but also correlated with a surgery that took place later in the week (Thursdays or Fridays).^13^ These studies, however, were not inclusive of all instrumented spine procedures. Instrumented fusion for cervical and lumbar pathology is among the most commonly conducted inpatient procedures, and it is this population that has an associated higher cost of care.^1,2,14^ An inclusive analysis will further elucidate the effect instrumented spine procedures the day the surgery takes place has on length of stay.

Additional predictors of increased length of stay include increased age, weekend admission, discharge to another facility, and any complications.^15^ Specifically for spine procedures, history of a nonspinal malignancy, history of pulmonary disease, cervical corpectomy, open as opposed to minimally invasive procedures, age, body mass index (BMI) greater than or equal to 40, American Society of Anesthesiology (ASA) physical status classification, multilevel procedures, surgical time, intraoperative transfusions or anemia, diabetes, and postoperative complications were notable predictors of an increased length of stay.^16,17,18,19,20^ A patient's comorbidities also play a role in length of stay. The ASA physical status classification and Charlson Comorbidity Index take into account the patient's medical history and are predictors of increases in complications rates.^21-23^ A higher ASA classification has been associated with an increase in length of stay and increased direct cost.^6,17,24^

The purpose of this study was to identify whether there were any differences in length of stay for instrumented spine procedures based on the day of the week the surgery took place. A secondary aim of the study was to determine whether the location of discharge to a rehabilitation facility would delay the discharge and whether there were any patient characteristics (age, sex, BMI, ASA classification, Charlson Comorbidity Index) associated with length of stay and discharge location.

## Methods

After obtaining approval from the institutional review board, a retrospective chart review was conducted using electronic medical records. The data included all instrumented spine cases done at a single academic tertiary center, in an inpatient hospital setting, by four orthopaedic spine surgeons, with greater than five years of attending surgical experience, from January 1, 2019, to December 31, 2019. All patients, regardless of sex, comorbid conditions, and age, who underwent an instrumented spine procedure during the selected study period were identified. All regions of the spine were included as long as they involved instrumentation (cervical, cervicothoracic, thoracic, thoracolumbar, lumbar, and sacral). A total of 791 patients were identified and divided into six groups corresponding to the day the surgery was conducted; there were no cases that met the inclusion criteria on Sunday. Patients were excluded if they underwent an instrumented fusion for spinal fracture, infection, or tumor. Patients younger than 18 years were also excluded from the study.

Demographic data (age, sex, BMI, ASA classification, and Charlson Comorbidity Index),^23^ the patient's day of surgery, discharge day, region of the spine, and discharge location (home with self-care, home with home health care, skilled nursing facility, or inpatient rehabilitation). Length of stay was defined as the number of midnights a patient stayed in the hospital after surgery. Patients admitted through the emergency department and who underwent an instrumented procedure were included, but their length of stay was based on the day they had surgery and not on their day of admission. Discharge readiness was determined based on clinical judgment of the admitting services. Discharge disposition was grouped into two cohorts: discharge home and discharge to a rehabilitation facility. The rehabilitation facility discharge included short-term nursing facility, subacute rehabilitation facility, and inpatient rehabilitation.

Bivariate analyses were used to examine the association of patient characteristics and each outcome of interest (hospital length of stay and discharge disposition). Continuous and categorical variables were expressed as means ± SDs along with medians (interquartile ranges) and frequencies (percentages), respectively. The assumption of normality of continuous variables was inspected by the Kolmogorov-Smirnov test. Owing to the potential skewness, the Kruskal-Wallis test with Dwass-Steel-Critchlow-Fligner multiple comparison (continuous variables) and chi square or Fisher exact test with multiple comparison by Bonferroni adjustment (categorical variables) were used to examine any difference. All tests were 2-sided, with a *P* value of < 0.05 considered to be statistically significant. Analyses were conducted using SAS v9.4 (SAS Institute).

## Results

**Figure 1:**
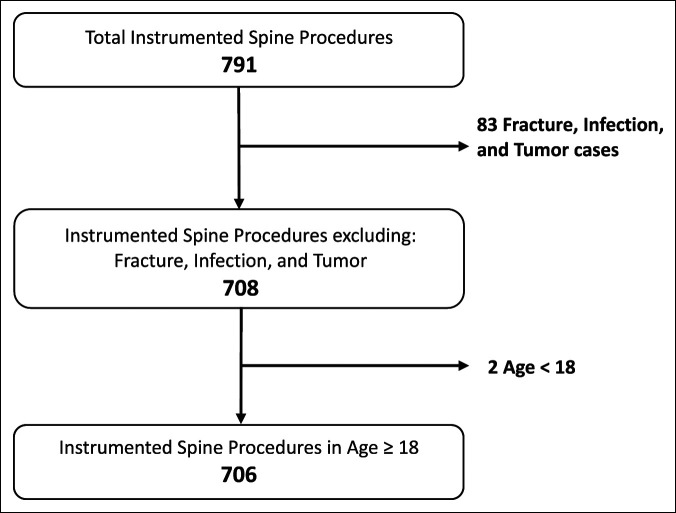
Flowchart showing a schematic of the patient population meeting inclusion criteria for analysis.

A total of 791 patients underwent an instrumented spine fusion from January 1, 2019, to December 31, 2019; 83 patients were excluded because of instrumented spine fusion for fracture, infection, or tumor; two patients were excluded because they were younger than 18 years at the time of the procedure. A total of 706 patients, therefore, were included in the analysis (Figure 1). These included 256 cervical, 11 cervicothoracic, 12 thoracic, eight thoracolumbar, 417 lumbar, and two sacroiliac joints. Preoperative determination for surgical intervention was at the discretion of the operating surgeon, all of whom primarily have a degenerative spine practice. Patients were grouped based on the day of their surgery: Monday (123, 17.4%), Tuesday (218, 30.9%), Wednesday (97, 13.7%), Thursday (180, 25.5%), Friday (72, 10.2%), or Saturday (16, 2.3%); there were no Sunday cases that met the inclusion criteria. There were 27 ACDF cases, two cervical disk arthroplasties, and two sacroiliac joint fusion cases that were discharged with a length of stay of zero; this accounted for 4.4% of the study population. Table 1 summarizes the distribution of age, sex, and BMI among the six groups. No differences in age or sex were observed between each of the groups. The BMI between the groups was significantly different (*P* < 0.05). The average BMI was 30.0 ± 5.8; lower than average BMI cases occurred on Fridays (28.4 ± 5.4) and Saturdays (28.4 ± 6.2), and higher than average BMI cases occurred on Wednesdays (31.0 ± 6.6).

**Table 1:** Patient Characteristics by Surgery Day

Variables^a^	Overall	Surgery Day						*P* Value
		Monday	Tuesday	Wednesday	Thursday	Friday	Saturday	
n	706	123 (17.4%)	218 (30.9%)	97 (13.7%)	180 (25.5%)	72 (10.2%)	16 (2.3%)	
Age (yr)	62.0 ± 12.3	62.3 ± 12.1	62.9 ± 11.9	61.7 ± 12.3	61.1 ± 12.9	63.0 ± 12.2	56.8 ± 14.1	0.39
	63.0 (54.0-71.0)	63.0 (55.0-71.0)	64.5 (55.0-72.0)	61.0 (55.0-71.0)	62.0 (54.0-70.0)	63.0 (54.5-72.0)	54.5 (50.5-67.5)	
18-40	47 (6.7%)	5 (4.1%)	11 (5.1%)	8 (8.3%)	18 (10.0%)	3 (4.2%)	2 (12.5%)	0.46
41-55	150 (21.3%)	30 (24.4%)	44 (20.2%)	17 (17.5%)	34 (18.9%)	18 (25.0%)	7 (43.8%)	
56-65	206 (29.2%)	30 (24.4%)	64 (29.4%)	32 (33.0%)	58 (32.2%)	19 (26.4%)	3 (18.8%)	
66-75	199 (28.2%)	39 (31.7%)	67 (30.7%)	28 (28.9%)	44 (24.4%)	19 (26.4%)	2 (12.5%)	
>75	104 (14.7%)	19 (15.5%)	32 (14.7%)	12 (12.4%)	26 (14.4%)	13 (18.1%)	2 (12.5%)	
Sex								
Male	312 (44.2%)	52 (42.3%)	95 (43.6%)	44 (45.4%)	80 (44.4%)	34 (47.2%)	7 (43.8%)	0.99
Female	394 (55.8%)	71 (57.7%)	123 (56.4%)	53 (54.5%)	100 (55.6%)	38 (52.8%)	9 (56.3%)	
BMI (kg/m^2^)	30.0 ± 5.8	30.3 ± 5.8	29.6 ± 5.8	31.0 ± 6.6	30.6 ± 5.4	28.4 ± 5.4	28.4 ± 6.2	0.02
	29.2 (25.8-33.4)	29.8 (26.4-33.8)	29.1 (25.6-32.9)	30.5 (26.6-34.1)	29.9 (26.6-33.9)	27.7 (24.3-31.9)	27.0 (24.8-29.5)	
<25.0	130 (18.4%)	21 (17.1%)	47 (21.6%)	15 (15.5%)	23 (12.8%)	20 (28.8%)	4 (25.0%)	0.01
25.0-30.0	246 (34.8%)	41 (33.3%)	72 (33.0%)	30 (30.9%)	69 (38.3%)	25 (34.7%)	9 (56.3%)	
30.1-35.0	199 (28.2%)	38 (30.9%)	59 (27.1%)	31 (32.0%)	48 (26.7%)	22 (30.6%)	1 (6.3%)	
35.1-40.0	89 (12.6%)	17 (13.8%)	28 (12.8%)	13 (13.4%)	30 (16.7%)	0 (0.0%)	1 (6.3%)	
≥40.1	42 (6.0%)	6 (4.9%)	12 (5.5%)	8 (8.3%)	10 (5.6%)	5 (6.9%)	1 (6.3%)	

BMI = body mass index

a For continuous variables, means ± SDs and medians (interquartile ranges) were presented. For categorical variables, frequencies (percentages) were presented.

The length of stay, on average, was 2.7 ± 3.7 days with a median of 2.0 (1.0 to 3.0) days (Table 2). A significant difference was observed when comparing the six days included (*P* = 0.004); however, the data were skewed because of Saturday surgeries (Table 2). When Saturday cases were excluded, there was no significant difference (*P* = 0.17) between the length of stay when comparing Monday, Tuesday, Wednesday, Thursday, and Friday surgery days (Table 2).

**Table 2:** Length of Stay by Surgery Day of the Week

Variable	Overall	Surgery Day						*P* Value
		Monday	Tuesday	Wednesday	Thursday	Friday	Saturday	
n	706	123 (17.4%)	218 (30.9%)	97 (13.7%)	180 (25.5%)	72 (10.2%)	16 (2.3%)	
LOS,^a^ d	2.7 ± 3.7	2.4 ± 2.9	2.7 ± 3.0	2.9 ± 6.0	2.5 ± 2.4	3.1 ± 4.0	5.8 ± 7.1	0.004
	2.0 (1.0-3.0)	1.0 (1.0-2.0)	2.0 (1.0-3.0)	2.0 (1.0-3.0)	2.0 (1.0-3.0)	2.0 (1.0-3.0)	3.0 (2.0-5.5)	
n^b^	690	123 (17.8%)	218 (31.6%)	97 (14.1%)	180 (26.1%)	72 (10.4%)	—	
LOS,^a,b^ d	2.7 ± 3.5	2.4 ± 2.9	2.7 ± 3.0	2.9 ± 6.0	2.5 ± 2.4	3.1 ± 4.0	—	0.17
	2.0 (1.0-3.0)	1.0 (1.0-2.0)	2.0 (1.0-3.0)	2.0 (1.0-3.0)	2.0 (1.0-3.0)	2.0 (1.0-3.0)	—	

LOS = length of stay

a For LOS, means ± SDs and medians (interquartile ranges) were presented.

b Sixteen patients who received surgery on Saturday were excluded on analysis.

Patient characteristics (age, sex, BMI, ASA classification, and Charlson Comorbidity Index) were individually analyzed for differences in length of stay (Table 3). Age, sex, ASA classification, and Charlson Comorbidity Index were significantly associated with length of stay (*P* < 0.001, *P* = 0.002, *P* < 0.001, and *P* < 0.001, respectively), whereas the patient's BMI was not (*P* = 0.77). Age, ASA classification, and Charlson Comorbidity Index were positively associated with length of stay (Spearman correlation coefficient: 0.23, 0.27, and 0.28, respectively; all *P* < 0.001). Furthermore, the multiple comparison analysis identified the notable pairwise difference to be between the 18- to 40-year-old age group to the 41- to 55-year-old age group, the 41- to 55-year-old age group to >75-year-old age group, the 56- to 65-year-old group to the age greater than 75, and the 66- to combined 41- to 55-year-old and great than 75-year-old group. The 18- to 40-year-old group represented 6.7% of the study population and the longest length of stay with an average of 4.5 ± 9.8 days (median 2.0 [1.0 to 3.0]). The older than 75 years group had the next longest average length of stay with an average of 3.3 ± 2.7 days (median 3.0 [2.0 to 4.0]). When compared with the total average length of stay, the older than 75 years group stayed 0.6 days longer. Women represented 55.8% of the study population and stayed, on average, 0.2 days longer than men, which was significant (*P* = 0.002). Patients with an ASA classification of 3 or 4 stayed significantly 1.6 days longer than the lower ASA classification of 1 or 2 (*P* < 0.001). Increased comorbidities, calculated by the Charlson Comorbidity Index, were associated with an increased length of stay (*P* < 0.001). Patients who were scored a 4 or greater on the Charlson Comorbidity Index had longer hospital stay on average (4.3 days ± 3.4, median 3.0 [2.0 to 6.0]) when compared with patients who had a score of 0 and 1 to 3, respectively (*P* < 0.001; *P* = 0.005). Similarly, patients who were scored zero on the Charlson Comorbidity Index had the shortest length of stay, on average 2.1 ± 3.4 days (median 1.0 [1.0 to 2.0]), which was significantly different when compared with a score of 1 to 3 and ≥4, respectively (*P* < 0.001; *P* < 0.001).

**Table 3:** Length of Stay by Patient Characteristics

Variables	n (%)	LOS^a^	*P* Value
Overall	706	2.7 ± 3.7; 2.0 (1.0-3.0)	
Age (yr)			
18-40	47 (6.7%)	4.5 ± 9.8; 2.0 (1.0-3.0)	<0.001
41-55	150 (21.3%)	2.1 ± 3.0; 1.0 (1.0-2.0)	
56-65	206 (29.2%)	2.4 ± 2.6; 2.0 (1.0-3.0)	
66-75	199 (28.3%)	2.8 ± 2.7; 2.0 (1.0-3.0)	
>75	104 (14.7%)	3.3 ± 2.7; 3.0 (2.0-4.0)	
Sex			
Male	312 (44.2%)	2.6 ± 4.3; 2.0 (1.0-3.0)	0.002
Female	394 (55.8%)	2.8 ± 3.2; 2.0 (1.0-3.0)	
BMI (kg/m^2^)			
<25.0	130 (18.4%)	2.7 ± 3.9; 2.0 (1.0-3.0)	0.77
25.0-30.0	246 (34.8%)	2.9 ± 4.7; 2.0 (1.0-3.0)	
30.1-35.0	199 (28.2%)	2.5 ± 2.1; 2.0 (1.0-3.0)	
35.1-40.0	89 (12.6%)	2.9 ± 3.3; 2.0 (1.0-3.0)	
≥40.1	42 (5.9%)	2.7 ± 2.5; 2.0 (1.0-3.0)	
ASA classification			
1-2	300 (42.5%)	1.8 ± 1.6; 1.0 (1.0-2.00)	<0.001
3-4	406 (57.5%)	3.4 ± 4.5; 2.0 (1.0-4.0)	
Charlson Comorbidity Index (disease only)			
0	369 (53.3%)	2.1 ± 3.4; 1.0 (1.0-2.0)	<0.001
1-3	293 (41.5%)	3.2 ± 3.9; 2.0 (1.0-3.0)	
≥4	44 (6.2%)	4.3 ± 3.4; 3.0 (2.0-6.0)	

ASA = American Society of Anesthesiology physical status, BMI = body mass index, LOS = length of stay

a For LOS, means ± SDs and medians (interquartile ranges) were presented.

Most patients (86.4%) were discharged home from their hospital stay (Table 4). On Saturday, 62.5% of patients were discharged home and 37.5% were discharged to a rehabilitation facility. When including Saturday, discharge home versus rehabilitation discharge demonstrated a significant difference (*P* = 0.03), but when comparing weekday cases only, there was no significant difference in discharge disposition (*P* = 0.36; Table 4). The highest percentage of patients who were discharged to a rehabilitation facility after a weekday case occurred on Fridays (19.4%), but this did not markedly affect the length of stay because there was no notable difference in length of stay when comparing surgeries that occurred during a weekday. Patients who were discharged to a rehabilitation facility had a significantly increased length of stay and were associated with an increased age, an increased ASA classification, and a Charlson Comorbidity Index of 1 or higher; but sex (*P* = 0.16) and BMI (*P* = 0.68) did not affect the patient's disposition status (Table 5). The length of stay for patients discharged to rehabilitation was 6.8 ± 5.3 days (median 5.5 [4.0 to 8.0]) and those patients discharged home was 2.1 ± 2.9 days (median 2.0 [1.0 to 2.0], *P* < 0.001). Of the patients who were discharged to a rehabilitation facility, 35.4% were 66 to 75 years and 31.3% were older than 75 years; resulting 66.7% of the patients who were discharged to a rehabilitation facility were older than 66 years. The pairwise comparison indicated that there was a significant difference when comparing the 41- to 55-year-old age group with the 66- to 75-year-old age group (*P* = 0.009) and older than 75 years age group (*P* < 0.001), respectively, and comparing the 66- to 75-year-old age group with the older than 75 years age group (*P* < 0.001). An ASA classification of 3 to 4 significantly increased the patient's discharge to a rehabilitation facility; 85.4% of patients discharged to a rehabilitation facility were in this ASA classification range (*P* < 0.001). The highest percentage of rehabilitation discharges had a Charlson Comorbidity Index of 1 to 3 (54.2%), and this was significant when comparing patients with a Charlson Comorbidity Index of 0 to 1 to 3 and ≥4, respectively (*P* < 0.001; *P* < 0.001). Only 18.7% of patients discharged to a rehabilitation facility had 4 or greater Charlson Comorbidity Index in this study.

**Table 4:** Discharge Disposition by Surgery Day of the Week

Variable	Overall	Surgery Day						*P* Value
		Monday	Tuesday	Wednesday	Thursday	Friday	Saturday	
n	706	123 (17.4%)	218 (30.9%)	97 (13.7%)	180 (25.5%)	72 (10.2%)	16 (2.3%)	
Discharge disposition^a^								
Home	610 (86.4%)	105 (85.4%)	194 (89.0%)	87 (89.7%)	156 (86.7%)	58 (80.6%)	10 (62.5%)	0.03
Rehabilitation	96 (13.6%)	18 (14.6%)	24 (11.0%)	10 (10.3%)	24 (13.3%)	14 (19.4%)	6 (37.5%)	
n^b^	690	123 (17.8%)	218 (31.6%)	97 (14.1%)	180 (26.1%)	72 (10.4%)	—	
Discharge disposition^a,b^								
Home	610 (86.4%)	105 (85.4%)	194 (89.0%)	87 (89.7%)	156 (86.7%)	58 (80.6%)	—	0.36
Rehabilitation	96 (13.6%)	18 (14.6%)	24 (11.0%)	10 (10.3%)	24 (13.3%)	14 (19.4%)	—	

a Discharge home included home with self-care and home with health care; discharge to rehabilitation facility included short-term nursing facility, subacute rehabilitation facility, and inpatient rehabilitation. Frequencies (percentages) were presented.

b Sixteen patients who received surgery on Saturday were excluded on analysis.

**Table 5:** Patient Characteristics by Discharge Disposition

Variables^a^	n (%)	Discharge Disposition^b^		*P* Value
		Rehabilitation	Home	
LOS, d	706	6.8 ± 5.3	2.1 ± 2.9	<0.001
		5.5 (4.0-8.0)	2.0 (1.0-2.0)	
Age (years)				
18-40	47 (6.7%)	6 (6.3%)	41 (6.7%)	<0.001
41-55	150 (21.3%)	8 (8.3%)	142 (23.3%)	
56-65	206 (29.2%)	18 (18.7%)	188 (30.8%)	
66-75	199 (28.3%)	34 (35.4%)	164 (27.1%)	
>75	104 (14.7%)	30 (31.3%)	74 (12.1%)	
Sex				
Male	312 (44.2%)	60 (62.5%)	334 (54.8%)	0.16
Female	394 (55.8%)	36 (37.5%)	276 (45.2%)	
BMI (kg/m^2^)				
<25.0	130 (18.4%)	20 (20.8%)	110 (18.0%)	
25.0-30.0	246 (34.8%)	38 (39.6%)	208 (34.1%)	
30.1-35.0	199 (28.2%)	23 (24.0%)	176 (28.9%)	
35.1-40.0	89 (12.6%)	10 (10.4%)	79 (12.9%)	
≥40.1	42 (5.9%)	5 (5.2%)	37 (6.1%)	
ASA classification				
1-2	300 (42.5%)	14 (14.6%)	286 (46.9%)	<0.001
3-4	406 (57.5%)	82 (85.4%)	324 (53.1%)	
Charlson Comorbidity Index (disease only)				
0	369 (53.3%)	26 (27.1%)	343 (56.2%)	<0.001
1-3	293 (41.5%)	52 (54.2%)	241 (39.5%)	
≥4	44 (6.2%)	18 (18.7%)	26 (4.3%)	

ASA = American Society of Anesthesiology physical status, BMI = body mass index, LOS = length of stay

a Discharge home included home with self-care and home with health care; discharge to rehabilitation facility included short-term nursing facility, subacute rehabilitation facility, and inpatient rehabilitation.

b For LOS, means ± SDs and medians (interquartile ranges) were presented. For categorical variables, frequencies (percentages) were presented.

## Discussion

Length of hospital stay affects healthcare costs, and efforts to efficiently and safely discharge a patient will lower the cost burden. In the total joint arthroplasty literature, there is evidence that indicates a surgery that occurs later in the week will increase the patient's length of stay^4,5,6,7,8,9^ while only one publication on lumbar laminectomy for lumbar spinal stenosis was associated with a longer length of stay if the surgery was conducted later in the week.^13^ In ACDF and minimally invasive lumbar laminectomy without fusion procedures, length of stay was not affected by the day the surgery took place.^11,12^ It has not been published, however, whether the length of stay for patients who underwent an instrumented spine fusion would be affected by the surgical day because these patients typically spend at least one midnight in the hospital in contrast to ACDF and minimally invasive lumbar laminectomy. Our retrospective review of instrumented spine cases over the course of a single year, at a single institution, conducted by four orthopaedic spine surgeons identified that the day the surgery occurred did not affect the length of stay. The cases consisted primarily of elective cervical and lumbar procedures for degenerative pathology. While a Saturday case did result in a longer length of stay, isolating cases from Monday to Friday, there was no difference in the length of stay, consistent with the publications on ACDF and minimally invasive lumbar laminectomy surgeries. Although this is large, single-institution study that included 706 cases, a larger population may demonstrate differences in day of surgery affecting length of stay. The inherent limitations of a retrospective review of electronic medical records that may affect the length of stay were postoperative complications, intensive care unit admissions, number of levels instrumented, surgical time, and reasons for a patient to be transferred to a rehabilitation facility. In addition, a study including procedures for deformity, trauma, and/or tumor spine pathology would likely affect the results. The initial hypothesis was that surgeries conducted later in the week would have a longer length of stay. It should be noted, however, that more cases were conducted early in the week. If defining early in the week as Monday, Tuesday, and Wednesday, this equaled to 62% of the cases in this study versus later in the week, defined as Thursday and Friday, 35.7%, with 10.2% of the total cases occurring on Fridays. Despite the limitations of this study, the data collected for a heterogeneous population demonstrated no difference in length of stay based on the day of the week.

Many surgical factors could also influence the length of stay if separately analyzed from the dataset presented. Namely, the statistical analysis included cases that had a length of stay of zero. These cases were primarily ACDF, one or two levels, that were discharged on the same day of the surgery. The specific day of the week that surgeries with a same-day discharge were conducted was not separated for analysis, and therefore, it is possible that the case makeup per day would influence the length of stay, for example, if more discharge on postoperative day 0 occurred on a specific day of the week. In addition, the analysis did not take into account the number of levels that were instrumented, which could affect the length of stay if the procedure included a larger dissection and, therefore, increased postoperative pain. The study data did not isolate for minimally invasive compared with open procedures, which has been associated with a trend toward reduced length of stay.^12,20^ Cases were also not separated into primary or revision procedures because revision lumbar spine procedures have been shown to have a mean length of stay of 6 ± 2.4 days in one study,^19^ which was higher than the average shown in our data of 2.7 ± 3.7 days.

Age older than 75 years, ASA 3 or 4, and increasing Charlson Comorbidity Index were associated with increased length of stay, whereas BMI did not affect length of stay; these results are consistent with existing publications.^5,6,9,15,17,18,24^ Although BMI may contribute to increased medical comorbidities, it did not affect the length of stay for instrumented spine cases analyzed in this study. The 18- to 40-year-old group demonstrated the longest length of stay, but this seems to be skewed by a small number of cases that had a prolonged length of stay because the SD was higher than the other groups and the median length of stay matched the median length of stay (2.0 days, 1.0 to 3.0 interquartile range) for the entire study population. The older than 75 years group, however, seems to be associated with an increased length of stay after an instrumented spine procedure, staying 0.6 days longer.

Many factors can contribute to the discharge efficiency in the postoperative period: clearance from physical therapy services, clearance from medical/ancillary services, acceptance from rehabilitation facilities, and/or insurance approval for discharge to a facility or assistance with home care setup. The data presented in this study indicated that age older than 66 years, ASA classification of 3 or 4, and Charlson Comorbidity Index of 1 to 3 will increase a patient's disposition to a rehabilitation facility and that those patients who were discharged to a rehabilitation facility had an increased length of stay, on average 4.7 days longer. However, this did not correlate with the day of the week (early compared with late) as has been previously published for a lumbar laminectomy.^13^ Knowing these factors that influence the length of stay, a patient can be more efficiently discharged to a rehabilitation facility. Surprisingly, a Charlson Comorbidity Index of 4 or more was associated with an increased hospital stay. Perhaps these patients required a longer length of stay so that they may be medically optimized before being discharged home.

## Conclusion

This study further strengthens the argument that day of surgery does not influence length of stay for patients undergoing an instrumented spine fusion. Age older than 75 year, female, ASA classification of 3 or 4, and increasing Charlson Comorbidity Index all influenced length of stay for instrumented spine fusions while BMI did not. Most instrumented spine fusion patients were able to be safely discharged home. Patients older than 66 years and with an ASA classification of 3 or 4 and a Charlson Comorbidity Index of 1 to 3 were associated with a discharge to a rehabilitation facility, and patients who were discharged to a rehabilitation facility had a longer length of stay. Identifying patients who are at risk of being discharged to a rehabilitation facility may assist in a more efficient disposition to decrease their length of stay and, therefore, the hospital cost of surgery.
